# Practical Uses and Advantages

**Published:** 1921-03-26

**Authors:** 


### DRIED MILK.

### II. Practical Uses and Advantages.

By A MEDICAL OFFICER OF HEALTH.

In the first article we gave some account of the history of dried milk and the methods of manufacture, and we now propose to consider it from the standpoints of bacteriology, vitamines, infant feeding, and general utility.

### The Bacteriological Standpoint.

From a bacteriological standpoint our information is not very complete, but such examinations as have been made show, as we should expect from the fact that it is subjected to considerable heat during manufacture, that it is much purer than ordinary milk. Most of the ordinary bacteria are killed, though some sporing organisms seem to survive owing to their greater resistance. But from a practical point of view the most important fact is that tubercle bacilli have never been found, and it is concluded that they are either killed or rendered so harmless as not to infect guinea-pigs. This is in itself most important, and raises its value in this respect over ordinary commercial raw milk, in which the number of samples taken show tubercle bacilli in 10 to 20 per cent. of those examined. When one considers the ravages of tubercle due to milk infection the importance of this cannot be over-estimated.

### The Vitamine Content.

Coming next to vitamines, we need consider only those, the absence of which cause rickets and scurvy. The former is known as the anti-rachitic and the latter as the anti-scorbutic vitamine. The advocates of raw milk, both from the breast and the cow, maintain that boiling and other forms of heating either partially or wholly destroy its "living" qualities, meaning by this those indefinable substances which are the necessary adjuncts of all foods, especially for growing mammals; but experiments as well as practical experience have proved these fears to be unfounded. In some towns, such as Leicester, which has been feeding many infants from birth upwards on dried milk for ten or twelve years past, it is found that both scurvy and rickets are extremely rare amongst children fed on dried milk—one might almost say as rare as among breast-fed children, and when they occur can generally be attributed to either too much dilution of the milk, digestive trouble, or some other insanitary condition associated with these diseases. The writer has also seen a very large number of children fed on dried milk, and has never seen a case of either of these diseases which could be definitely stated to be due to the use of dried milk. One may therefore conclude that the anti-scorbutic and the anti-rachitic vitamines are at least not wholly destroyed, but remain in sufficient quantities to prevent the corresponding diseases.

There is another advantage over raw cow's milk, and that is that the curd formed in the stomach from dried milk is fine and flocculent, thus somewhat resembling that formed from human milk, and this accounts for the fact that dried milk often agrees with delicate children when many other artificial foods have failed.

### Two Manufactured Grades.

Dried milks are often made in two kinds, according to the amount of cream present, known as full and half-cream milks, but it is shown in the L.G.B. report referred to above, and practical experience also proves that this division is not only unnecessary but may prove harmful. Practically all infants can quite well digest the full cream milk, and they thrive much better on it than on the half-cream powder. It is always better in the case at least of healthy infants to overfeed with fat than underfeed. Some physicians recommend that a certain amount of orange juice should be taken, while the infant depends on dried milk alone. It can certainly do no harm, but here again practical experience has shown this precaution to be unnecessary since scurvy, as mentioned above, is very rare in infants fed on dried milk.

### General Utility.

Many other advantages could be mentioned, but the last we will quote, and in our opinion the most important, is its usefulness as an infant food during the hot summer months. Everyone is aware of the enormous mortality among bottle-fed babies from summer diarrhœa—how the milk even when boiled becomes contaminated in the home from dirty vessels, flies and dust, &c., to the detriment of the infant, but this can always be avoided by making the infant's milk fresh for each feed from dried milk, and making as nearly as possible the exact amount required, any that is left being thrown away or better given to the domestic animals which are always about the home. One word of warning remains as to dilution. The tendency in most cases seems to be to dilute the powder too freely, and if this is done the infant will not flourish, but as a rule directions will be found on the packet which show the amount of powder and water required to form ordinary milk. Each part of dried milk powder mixed with seven parts by weight of water will form ordinary milk, *i.e.*, a little under six ounces to one quart. As to price it is certainly no dearer than ordinary milk and many maintain that it works out a little cheaper. Weighing, therefore, the advantages and disadvantages, and considering that by drying milk we can draw our supplies from the remotest corners of the earth, I think a little more attention might be given to the product for the feeding of both adults and children.

The first article appeared on March 12, p. 535.

